# Patented technologies for schistosomiasis control and prevention filed by Chinese applicants

**DOI:** 10.1186/s40249-021-00869-6

**Published:** 2021-06-12

**Authors:** Yan-Hong Xiong, Xue-Nian Xu, Bin Zheng

**Affiliations:** National Institute of Parasitic Diseases, Chinese Center for Disease Control and Prevention, Chinese Center for Tropical Diseases Research, Key Laboratory of Parasite and Vector Biology, National Health Commission, WHO Centre for Tropical Diseases, National Center for International Research On Tropical Diseases, Ministry of Science and Technology, Shanghai, 200025 China

**Keywords:** Schistosomiasis, Patent analysis, Control and prevention, Technology

## Abstract

**Background:**

Many valuable and productive patented technologies have been developed to control schistosomiasis in China in the past 70 years. We conducted a research to analyse patented technologies for schistosomiasis control and prevention filed by Chinese applicants for determining the future patent layout.

**Methods:**

The patent databases of China National Intellectual Property Administration and Baiten were comprehensively searched, and patented technologies for schistosomiasis control and prevention, published between January 1950 and December 2020 filed by Chinese applicants were sorted on 30 December 2020. The patent types, technical fields, and patent development trends were analysed using patent indexing.

**Results:**

There are 184 valid schistosomiasis control technology patents, among them 128 invention patents. The patents related to schistosomiasis control and prevention technology have gone through the germination, growth, and maturity stages. These phases correspond with three phases in schistosomiasis control in China. The main technical aspects were fundamental research (*n* = 37), detection (*n* = 13), chemotherapy (*n* = 61), and armamentarium/devices (*n* = 73), of which the number of patents for detection for diagnosis was smaller. The top three specialised technical fields for patents subgroups, focusing on antiparasitic agents, DNA or RNA, vectors and medicines, of which schistosomicides are the major dominant subgroup.

**Conclusions:**

We recommend that technologies to be patented for schistosomiasis control and prevention be focused on detection, preliminary studies for molecular detection methods should be significantly enhanced, and patent layout must be performed, which will, in turn, promote accuracy of early diagnosis, not only in humans but also in livestock. It is necessary to develop more anti-schistosomal drugs safely and effectively, exceptionally eco-friendly molluscicides and herbal extracts anti-schistosomes, improve treatment, develop vaccines for use in humans.

**Graphic abstract:**

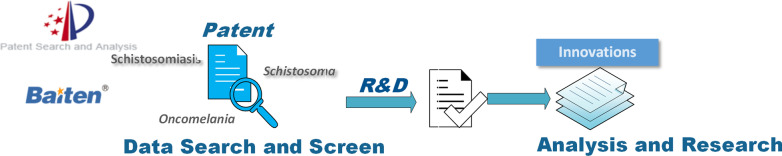

**Supplementary Information:**

The online version contains supplementary material available at 10.1186/s40249-021-00869-6.

## Background

Schistosomiasis is a zoonotic parasitic disease caused by six species of trematodes. The major three species of schistosomes, namely, *Schistosoma japonicum, S. manosni,* and *S. haematobium* cause schistosomiasis in a wide range of endemic areas and severe harm to human health [1–3]. According to World Health Organization (WHO) reports, approximately 779 million people worldwide have been infected with schistosomes, with 236.6 million cases in 2019 [4]. Schistosomiasis japonica caused by *S. japonicum* is predominantly prevalent in China [5–8]. A total of 30 170 advanced schistosomiasis cases was documented in China in 2019. *Oncomelania hupensis*, is the only intermediate host snail of *S. japonica*, with snail habitats 3.6 billion m^2^ in 2019 [9].

China has a 70-year-old history of schistosomiasis control. The national control programmes have achieved great success, and the endemic status of schistosomiasis in China is already low [10–14]. The technologies for schistosomiasis immunodiagnosis, chemotherapy, snail control, devices, and vaccines have been gradually developed, sanitation (faecal disposal) has been improved, and patents have been obtained to protect technological innovation [15–20]. However, many challenges in eliminating schistosomiasis exist [14]. Precision control of schistosomiasis still needs to be reinforced in China [9]. It's necessary to conduct a research on patented technologies for further patent payout.

In this study, we searched and analysed patent information on schistosomiasis control and prevention from the patent databases of the State Intellectual Property Office and Baiten to understand the developmental trends of schistosomiasis control and prevention in patents, analyze technical fields and patent layout. We present proposals for the structure of patented technologies, which may aid high-quality scientific and technological innovation [21] to achieve the national goal of eliminating schistosomiasis by 2030, as stated in the ‘Healthy China 2030’ Planning Outline [22].

## Methods

### Patent search strategy

The search was conducted in the China Patent Database from China National Intellectual Property Administration (CNIPA, weblink: https://www.cnipa.gov.cn/) and the Baiten Patent Database (weblink: https://www.baiten.cn/) independently for patents related to schistosomiasis control and prevention published between January 1950 and December 2020, using the following search terms: '*Schistosoma*' (all fields) AND '*Oncomelania*' (all fields) AND '*Schistosoma*' (patent specification) on December 30, 2020. We only included patents filed by Chinese applicants. We also undertook an additional manual filtration by abstracts and specifications of the patents and categorised the legal status.

### Study selection criteria

Only applications with patent titles were screened in the two databases with the same inclusion and exclusion criteria applied to the search strategy [23], and the duplicates were identified and deleted by using web clips in databases. We excluded foreign patents which were not filed by Chinese applicants, but passed the Patent Cooperation Treaty (PCT) into China. The patent literature was downloaded for further analysis. We deleted unrelated patents with detailed information and retained only valid patents as target patents among the total patents with three legal statuses, i.e. valid patents, invalid patents and patents under trial [24].

### Data extraction and statistical methods

According to the selection criteria mentioned above, the valid patents were extracted for analysis by patent analysis methods, and the technical domains of valid patents were analysed according to the International Patent Classification (IPC) number, the most common hierarchical system of categories used in the world [23, 25, 26]. Microsoft Excel (Office professional plus 2016, Microsoft, Redmond, USA) was used as the statistical analysis software to analyse the collected data [27].

## Results

**Fig. 1:**
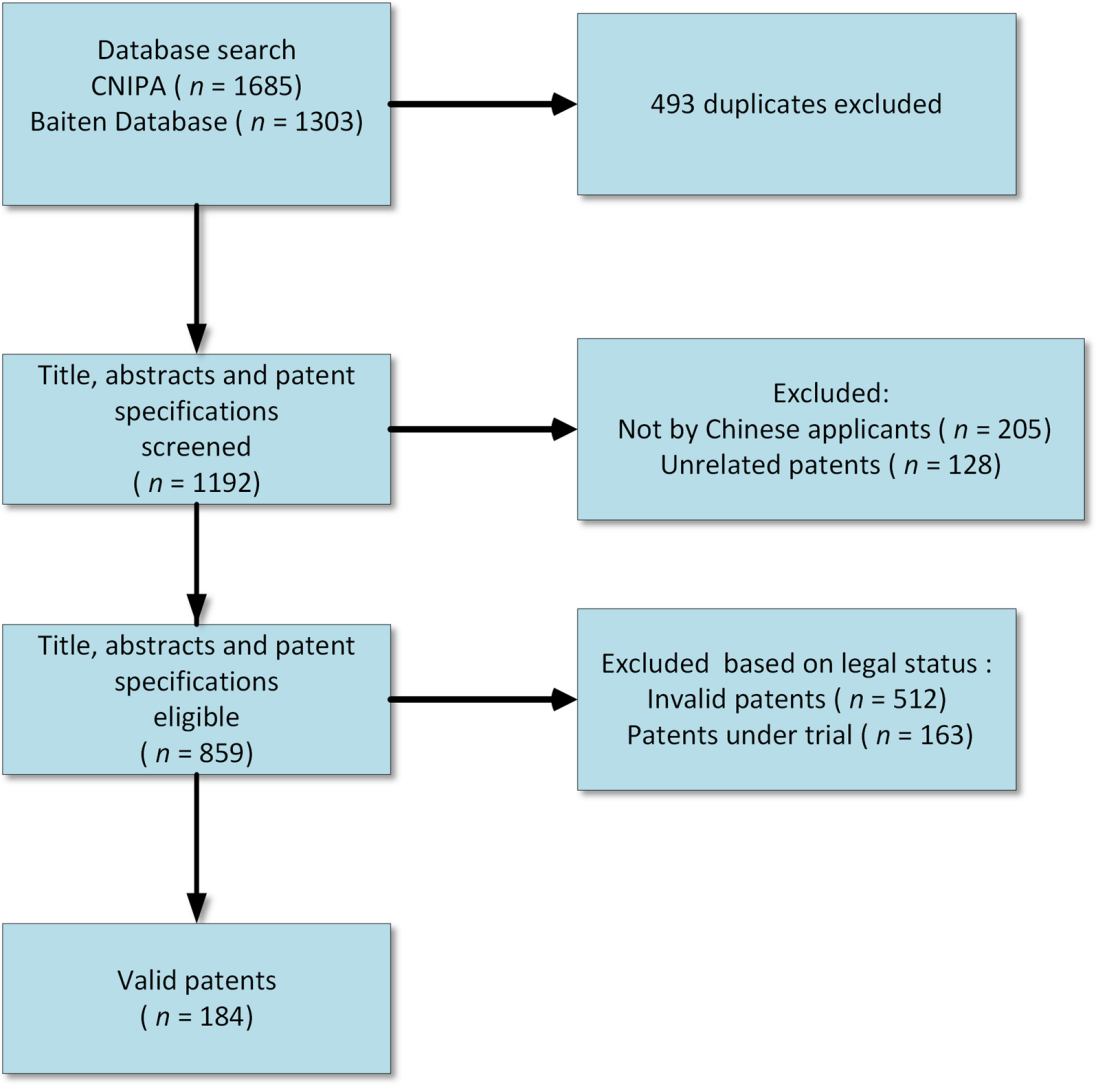
Flow chart of included patents. *CNIPA* China Patent Database from China National Intellectual Property Administration

By the study selection criteria and data extraction, 382 duplicates in CNIPA database were identified and deleted. Several patents had been repeated in '*Schistosoma*' (all fields) AND '*Oncomelania*' (all fields) AND '*Schistosoma*' (patent specification). We eliminated 111 duplicates using web clips in databases and excluded 205 patents according to the International Application Number. We deleted 128 unrelated patents and retained 184 valid patents as target patents among the 859 total patents filed by Chinese applicants (Fig. 1). A list of targeted patents is provided in Additional file 1.

### Overall trend analysis

**Fig. 2:**
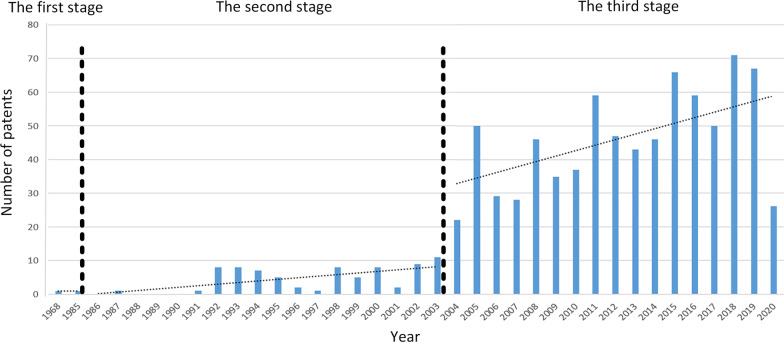
The number of patent applications between 1968 to 2020. The first stage (from 1950 to 1985): the germination period; The second stage (from 1986 to 2003): the growth period; The third stage (2004 onwards): the maturity period

According to the data obtained, patent applications by the Chinese for schistosomiasis control and prevention technologies began in 1968 (Fig. 2). After the stages of germination, growth, and maturity, the entire research and development process, exploration, and application have been carried out for schistosomiasis-related control technologies. Specifically, there was slow progress in the development of schistosomiasis-related technologies from 1950 to 1985, with only two patents, which were still undergoing research and investigation, being introduced in this period. From 1986 to 2003, the number of patent applications increased, with some of the critical technologies related to snail control, chemotherapy, and usage of proteins and antibodies, but the overall rate of application was still low. Since 2004, there has been rapid development in schistosomiasis-related technologies. The number of patent applications increased rapidly year-on-year and peaked in 2018, with 71 patent applications on the application-oriented research and development process. A list of the number of patents applications is provided in Additional file 2.

### Analysis of the legal status of patents

There are 184 valid patents, 512 invalid patents, and 163 patents under trial. A toal of 219 were invalid as a result of the termination of patent rights, and 214 of which were mainly due to unpaid annual fees, except 5 expired patents.

**Fig. 3:**
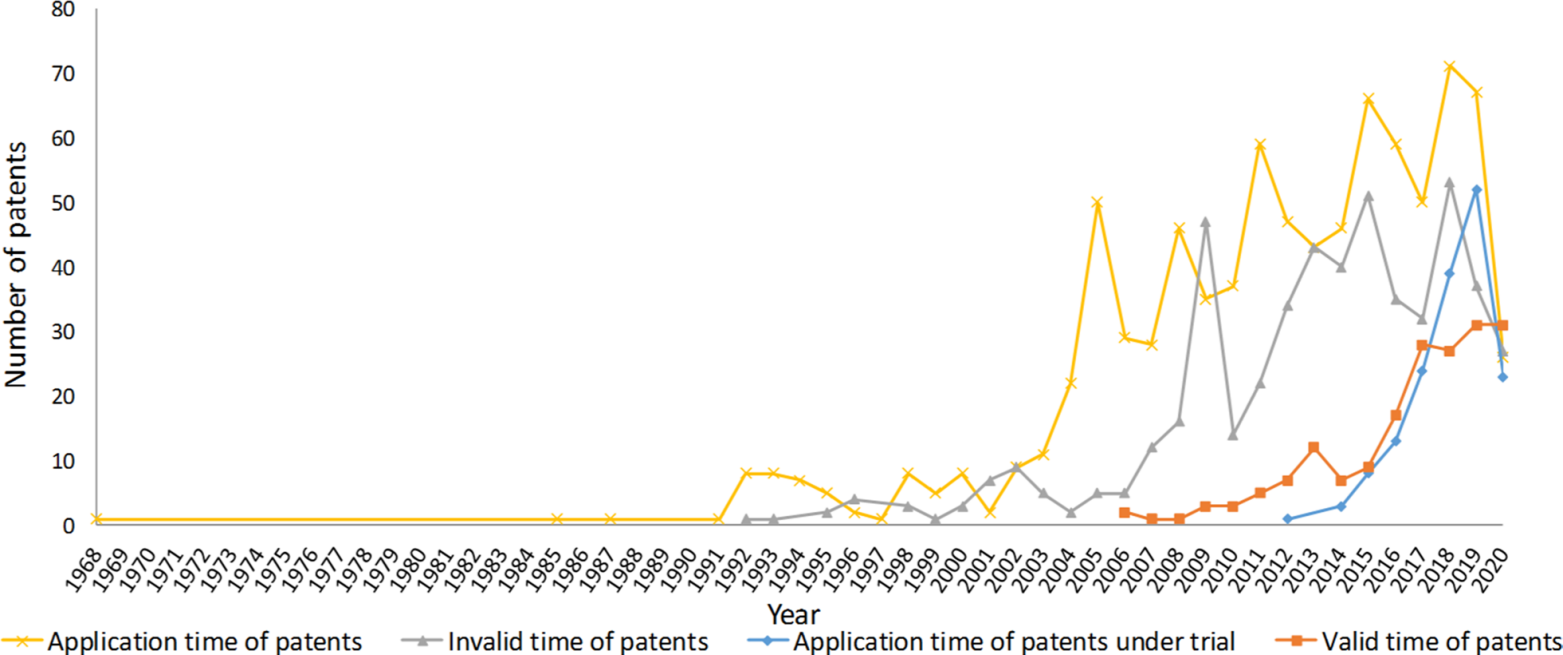
The number of patent applications, invalid patents, patents under trial, and valid patents during 1968–2020

As shown in Fig. 3, there was a substantial increase in the total number of patent activities between 1968 and 2020, including the overall number of patent applications, invalid patents, valid patents, and patents under trial. However, actual trends fluctuated, followed by several years of stagnation before the 1990s, mainly because of the relatively small number of applications. Patent activities increased significantly since 2004. In 2019, the total number of patent applications were 67, 52 under trial, 31 valid patents, and 37 invalid patents, with comparatively better results than in other years.

### Analysis of valid patents

#### Patent types

Most of the valid patents (69.6%, 128/184) are invention patents. And they mainly consist of fundamental research and pharmaceutical technologies, with 56 utility models accounting for 30.4% of valid patents.

By analyzing the types of applicants, part of the patents is jointly applied, among which 59 patents are applied jointly by colleges and universities and 91 patents by scientific research institutions, i.e., a total of 150 patents are involved, accounting for the vast majority; enterprises apply for 38 patents, and individuals apply for only 8 patents. Generally speaking, the patents are mainly applied by scientific research institutions.

#### Patent indexing analysis

Among the valid patents for the control and prevention of schistosomiasis, the major ones are related to schistosomiasis japonica, which is prevalent in China, and only one patent is about schistosomiasis haematobium. The necessary technical aspects are fundamental research (37, 20.1%), detection methods (13, 7.1%), and chemotherapy (61, 33.1%), mainly focused on armamentarium and devices (73, 39.7%). Detection technologies are relatively small in number, as presented in Table 1. For more detailed information, see the Additional file 1.

**Table 1:** Technology aspects of 184 valid patented technologies for schistosomiasis control and prevention

Technology aspects		Patented technology aspects
Fundamental research (*n* = 37, 20.1%)	*Oncomelania hupensis* (*n* = 1)	Detection: Specific gene: mitochondrial genome DNA to study genetic variation and differentiation of *O. hupensis* (*n* = 1)
	*Schistosoma japonicum* (*n* = 36)^a^	Detection methods (*n* = 17) Proteins: SJ16, SjSap, SjScP27, SjScP80, SjScP84, SjScP88, SjHSP90,Vamp2, Microsatellite DNA,nucleic acid,Schistosoma egg crude antigen, amino acid, nanoantibodies, transposon DNA target sequences, glutathione-S-transferase(GST), fluorescent markers
		Chemotherapy (*n* = 17) SJ16, SjP40, SjSAPLP4, SjSAPLP5, SjScP27, SjScP80, SjScP84, SjScP88, SjHSP60, IRF4, Frizzled 9, wingless/integrated (WNT) signaling pathways, Sj thioredoxin glutathione reductase (GR), Ad-TD-gene, O-GlcNAcylation, miRNAs, worm production
		Vaccine (*n* = 11) SJ16, Sj29, Sj23, SjE16, Sjp50, SjCTRL, SjSAPLP4, SjSAPLP5, SjScP27, SjScP80, SjScP84, SjScP88, SJ26 glutathione-S-transferase(GST), worm production
Detection methods (*n* = 13, 7.1%)^b^	Molecular detection methods (*n* = 8) *O. hupensis* (*n* = 2)/*S. japonicum* (*n* = 6)	LAMP technique (*n* = 3)
		PCR technique (*n* = 4)
		RPA technique (*n* = 1)
	Serological detection methods for *S. japonicum* (*n* = 5)	Electrochemical immunosensor (*n* = 1)
		ELISA technique (*n* = 2)
		GICA (*n* = 2)
	Pathogenic detection methods for *S. japonicum* (*n* = 1)	Improved Kato-Katz (*n* = 1)
Chemotherapy (*n* = 61, 33.1%)	Molluscicides for *O. hupensis* (*n* = 26)	Streptomyces (*n* = 3)
		Niclosamide (*n* = 6)
		Other chemical compounds or compounded with plant or nanocomposite (*n* = 17)
	Schistosomicides for *S. japonicum* (*n* = 35)	Praziquantel (*n* = 4)
		Artemisinin/Artesunate (*n* = 3)
		Herbal effective ingredient extracts (*n* = 12) Bo Luohui/Pulsatilla/Xusuizi/betel nut/Chinese medicine combination
		WNT and other chemical compounds (*n* = 15)
		External use with gossypol (*n* = 1)
Armamentarium/devices (*n* = 73, 39.7%)	For *O.* *hupensis* (*n* = 34)	Prevention of spread of snails/water conservation and electricity (*n* = 10)
		Molluscicidal device/crushing of *O. hupensis* (*n* = 7)
		*O. hupensis* search/collection (*n* = 10)
		*O. hupensis* tracking and raising/bioimaging/monitoring and testing (*n* = 7)
	For *S. japonicum* (*n* = 38)	Monitoring/Observation for cercariae/miracidium (*n* = 5)
		Incubation collection and separation research for cercariae/miracidium (*n* = 4)
		Ultrasonic larviciding (*n* = 1)
		Device for separating, hatching, and killing *S. japonicum* eggs (*n* = 3)
		Neck sleeve deinsectization device for removing schistosomes on necks of dogs (*n* = 1)
		Oral drug delivery device for cattle, sheep, and dogs (*n* = 3)
		Epidermal needle for the vaccine (*n* = 1)
		Detoxification and collection of excrement (*n* = 4)
		Kit devices for on-site test and laboratory analysis for schistosomiasis control and prevention (*n* = 16)
	For *S. haematobium* (*n* = 1)	Device of a detection filter membrane for filtering *S. haematobium* eggs (*n* = 1)

^a^The overlap of classification for partial patents of detection methods, chemotherapy and vaccine. ^b^One of the detection patents is based on protein Sjp7 for both molecular detection and serological detection, with 14 detection methods in 13 patents

*GST* Glutathione-*S*-transferase, *GR* Glutathione reductase, *LAMP* Loop-mediated isothermal amplification, *PCR* Polymerase chain reaction, *RPA* Recombinase polymerase amplification, *ELISA* Enzyme-linked immunosorbent assay, *GICA* Eolloidal gold immunochromatographic assay

Fundamental research involves the extraction of specific mitochondrial genome DNA from *O. hupensis* (*n* = 1) for detection, and development of technologies for *S. japonicum* (*n* = 36), involving antigen genes, proteins, or recombinant proteins, miRNAs, transposon DNA target sequences, glutathione-S-transferase (GST), glutathione reductase (GR), nanoantibodies, wingless/integrated (WNT) signaling pathways, nucleic acid, O-GlcNAcylation, and worm production, etc., which applying detection methods (*n* = 17), chemotherapy (*n* = 17), vaccine (*n* = 11), respectively, with the overlap of classification for partial patents.

A total of 14 detection methods have been introduced in 13 patents, which include molecular detection methods (*n* = 8), and serological detection methods (*n* = 5), and only one pathogen detection method involving improved Kato-Katz. The molecular detection methods described in the applications include loop-mediated isothermal amplification (LAMP) kit, polymerase chain reaction (PCR) kit, and recombinase polymerase amplification (RPA) kit for the detection *S. japonicum* nucleic acid. The serological detection methods described primarily include the schistosomiasis electrochemical immunosensor, enzyme-linked immunosorbent assay (ELISA) kit to detect *S. japonicum* antibodies, colloidal gold immunochromatographic assay (GICA) to detect antigens and antibodies, and so on. There are 2 for *O. hupensis* (LAMP and PCR) and 12 for *S. japonicum.*

Chemotherapy was implemented via molluscicides (*n* = 26) and schistosomicides (*n* = 35). The introduction of *Streptomyces*, salicylamide esters, methyl pyridine phosphorus, chlorphenoxyacetic acid, nicotinamide, tetramethyl acetaldehyde, methyl naphthol aldicarb, chlorothalonil, macrolides nitrolime, calcium cyanamide or compounded with plant or nanocomposite as molluscicides for snail control excepted niclosamide. In addition to the highly effective praziquantel, artemisinin and artesunate as anti-schistosomal drugs, schistosomicides also include several effective herbal extracts, heterocyclic compounds, α-tocopherol, calcium cyanamide, 2-[2-(4-chloro-2-nitrophenyl)diaz-1-enyl]-4,6-difluorophenol, 1,2,5-thiadiazole-2 oxide, 1,2,5-oxadiazole-2 oxide, 4-benzyl-1-phenethyl-piperazine-2,6-dione, drug for the treatment of liver fibrosis, and so on. The external use of gossypol is also effective in this regard. In total, two cercaricides include *N*-(4-nitrophenyl)-5-chlorosalicylamide and supramolecular hydrogel with *N*,*N'*-diaspartic acid-perylene tetracarboxylic diimide, and tetrahydrofuran.

Most current patents are for armamentarium and devices for the control of *O. hupensis* (*n* = 34), *S. japonicum* (*n* = 38), and *S. haematobium *(*n* = 1), these technologies are related to preventing the spread of snails, molluscicidal armamentarium, the crushing of *O. hupensis*, search, collection, tracking, raising, bioimaging, monitoring and testing for *O. hupensis*, testing related equipment; monitoring, observation, incubation collection, and separation research, ultrasonic larviciding associated with cercariae and miracidium, the device for separating, hatching and killing and filtering schistosome eggs, neck sleeve deinsectization device for removing schistosomes on necks of dogs; oral drug delivery devices for cattle, sheep, and dogs; an epidermal needle for a DNA vaccine against schistosomes; detoxification and collection of excrement; and kit devices for on-site test and laboratory analysis for schistosomiasis control and prevention.

### Analysis of technical field distribution

A61P33 (*n* = 45), C12N15 (*n* = 32), and A61K31(*n* = 26) are the top three dominant IPC subgroups for the technical field of schistosomiasis control, focusing on antiparasitic agents, DNA or RNA, vectors and medicines, of which A61P33/12, i.e., schistosomicides (*n* = 43) is the significant dominant subgroup by analyzing the technical field distribution of valid patents according to the IPC number.

## Discussion

### Analysis of patents in three phases in schistosomiasis control

The patented technologies related to schistosomiasis control and prevention have gone through three stages. These phases corresponded with three phases in schistosomiasis control in China: the first stage (transmission control strategy through snail control, from the mid-1950s to the early 1980s), the second stage (morbidity control based on chemotherapy, from the mid-1980s to 2003), and the third stage (integrated control strategy with an emphasis on infection source control, 2004 onwards) [6, 28], with the implementation of patents for interventions on infection source control, transmission control or transmission interruption, and protection of susceptible population [29]. Only two patents were applied in the first stage in China, which is the same as other outputs, such as articles. There were application patents with 77 pieces during the second stage, 30 (39.0%) related to snail control, and 13 (16.9%) related to chemotherapy. It was expected to summarise the previous stage's technologies, and thus there were significant patents that referred to snail control, the primary strategy in the first stage. During the third stage, the majority of patents (163) accompanied strategy under trial, which may become valid patents. All 184 valid patent applications were made after 2004. A total of 128 invention patents indicate more creativity with longer protective time than utility models. The number of patents has gradually increased. Through continuous research and development (R&D) and technological innovation, significant preliminary research results referring to genes, proteins, especially recombinant proteins, nucleotides, and pathways, with part of the primary research mentioned applied to multiple studies. Meanwhile, some devices used for experiments have become patents to promote basic research. The number of articles of schistosomiasis control strategy increased after 2004, with the same tendency for patents [30].

With the increasing number of imported schistosomiasis cases [31–37], there is currently a patented technology for producing a filter membrane for filtering *S. haematobium* eggs, and nine patents related to *S. manosni* and *S. haematobium* are under trial.

The technical field distribution from the analysis of IPC can also provide specific corroborative evidence to patent technologies. A study [24] found global patents for *Schistosoma* between 1985 and 2014. A similar application was also observed. The IPC classification of patents mainly concentrated around A61P33/12 (schistosomicides) and A61K39/00 (medicinal preparations containing antigens or antibodies). Patents on *Schistosoma* in China focused on A61P33, C12N15, and A61K31 after six years, of which A61P33/12 is the most common one. The technology subdomains are also pharmaceuticals and biotechnology.

It is necessary to carry out the patent layout for numerous invalid patented technologies for schistosomiasis control and prevention, especially the patent for which rights have been terminated without paying the annual fee (*n* = 214), with a high proportion of improper maintenance for a low commercial value [24]. We can explore technical points, actively pay attention to R&D trends, and seek technologies such as PCR and LAMP, etc., valuable detection and diagnosis technology points are patented.

### Patents for infection source control

There are 13 patents for detecting *S. japonicum* infection, including pathogenic diagnosis, serological diagnosis, and molecular diagnosis. Only one valid patent referred to the traditional pathogenic method (faecal examination: detecting schistosome eggs in faeces of infected source), the Kato-Katz technique as the 'golden method' to judge whether *Schistosoma* is infected or not. However, the pathogen-detecting method is effort-intensive, mainly it requires faecal sample collection, a long diagnosis cycle, and low sensitivity (especially in areas where the overall endemicity has become low), the high false-negative rate of approximately 5.56–89.47% [38–40].

There are five kit patents related to serological diagnosis, and many more patents in basic research targeted this kind of diagnosis. Serological diagnosis using four methods, that is, indirect haemagglutination assay (IHA), ELISA, dye dipstick immunoassay (DDIA), dot immunogold filtration assay (DIGFA), have simple operation and high sensitivity, and have the advantages of increased compliance with the people in epidemic areas; however, they cannot distinguish current infections from past ones. Such tests proved unsatisfactory specificity and were not suitable for early diagnosis [41, 42]. Currently, there are eight test kits approved by the National Medical Products Administration, that is, DDIA (*n* = 1), IHA (*n* = 3), ELISA (*n* = 2), and IHA (*n* = 2).

Eight kit patents referred to molecular diagnostic techniques, such as LAMP, RAP, PCR, etc., which have proven significant because of their speed, high specificity, and sensitivity. However, there is no molecular detection kit approved by the National Medical Products Administration, as they are relatively expensive, require a controlled environment, and are likely to cause false positives because of contamination. Inventions described in most of these types of patents are still used for humans and one for livestock. However, domestic animals are also the primary source of infection, and relevant testing products are urgently needed.

Generally, the diagnostic options and related products are still few, especially kits for detecting schistosomiasis. A more efficient, convenient, and rapid kit is urgently required to facilitate a more sensitive and rapid diagnosis of schistosomiasis.

The intervention approaches also included chemotherapy for humans and livestock. The large-scale deployment of praziquantel to control schistosomiasis in China significantly reduced morbidity due to *S. japonicum* [43, 44]. To date, there are only four valid patents regarding praziquantel formulations and compounds. Three patents related to artemisinin and its derivatives (artemether and artesunate) could have anti-schistosomal properties [45]. Many patents about new chemotherapy are mainly associated with in vitro trials and effective herbal extracts for anti-schistosomsis; however, the National Medical Products Administration has approved praziquantel among 19 products as the only therapeutic drug. Giving a green passageway to speed up approval of the adaptation of older drugs artesunate and artemisinin for anti-schistosomiasis will aid in controlling schistosomiasis.

It is challenging to control livestock, an infectious source that plays a crucial role in schistosomiasis transmission, as a primary reservoir host [41]. Patented technologies for oral drug delivery devices for cattle and sheep have emerged. After control and prevention measures, infectivity in cattle has been basically controlled, but sheep have gradually shown increasing infectivity and have become the primary infection source. One reason is that sheep dung, which is scattered everywhere and is not easy to collect. The use of a patented sheep dung collection device has been promoted, and other patented technologies such as equipment for the harmless treatment of faeces, washroom pan, etc., have been used for faeces management. Finally, faecal matter with infected eggs is being prevented from contaminating water sources to cut off the transmission.

### Patents for transmission control or transmission interruption

*O. hupensis*, the only intermediate host of *S. japonicum*, is not easy to control. The snail habitats in endemic regions are enormous, with approximately 3.6 billion m^2^ [9, 46, 47], a large majority located in the lake regions. With the restoration and protection of the ecological environment, many factors, such as temperature, rainfall, vegetation, and soil moisture, have also increased snail spreading. Thus, mass snail elimination campaigns have been developed for schistosomiasis control [41]. There are many patented technologies for snail management, and it is essential to apply scientific and technological strategies, such as a drainage system, and armamentarium for *O. hupensis*, and molluscicides to control snails. Patented snail control technologies that have low toxicity and are environmentally friendly [48], and effective snail surveys also have market application prospects.

One of the other primary strategies for snail control is chemotherapy. Niclosamide, the only approved molluscicide, is the most widely used in China [49], and has six patents, including patents for powder formulations, suspension concentrate formulations, and spreading oil formulations. Several new chemical compounds have been developed for snail control. Metaldehyde has excellent molluscicidal effects against *O. hupensis* and has low toxicity [44]. Calcium cyanamide (CaCN_2_) is not only a molluscicide but also can be used to kill *S. japonicum* eggs. It also acts as a nitrogen fertiliser and is both economical and environment-friendly. At present, further investigation is required for broader applications in the field. Several molluscicides and schistosomicides from herb extracts have been developed for patenting, which could potentially be safer. The government has been recommended to increase investment and provide supportive policy innovation services to promote effective herbal extracts.

### Patents for susceptible population protection

The development of the patented schistosomiasis vaccine is an adjuvant measure of strategic significance; it protects the susceptible population and helps in the integrated control of schistosomiasis. WHO Special Programme for Research and Training in Tropical Diseases (TDR) has placed the development of schistosomiasis vaccines at the forefront of research on the control and prevention of schistosomiasis. Currently, the schistosomiasis vaccine is mainly used in animals. More clinical trials for determining safety and efficacy in humans are required [50, 51].

### Limitations

This study has several limitations. First, patent retrieval is a complicated process, and it is challenging to identify targeted patents in online Chinese patent databases completely. Second, patent databases have drawbacks in terms of data collection. As databases have different retrieval standards, although the exact keywords were used, the results have significant differences. It is likely that this introduced some personal bias, as the system cannot automatically and accurately filter data.

## Conclusions

Patented technologies for schistosomiasis control and prevention have been researched and developed as integrated control strategies, transitioning to precise control, which has proven useful and has played a specific role in schistosomiasis control and prevention in China. However, there are many significant challenges; therefore, it is necessary to carry out R&D for precise technology and more advanced techniques with continuous innovation. Exploring technical applications, pursuing essential research on molecular detection, and applying preliminary results for detection while patenting the technologies can improve the accuracy of early diagnosis, not only in human beings but also in livestock, and patent layout must be performed. Safe and effective drugs for *S. japonicum* and *O. hupensis* still remain to be developed, exceptionally eco-friendly molluscicides and herbal extract anti-schistosomes. In addition, strategies for sufficient treatment and vaccines for human beings should be developed for better control. And patent layout must be performed. Technologies worthy of mention are those related to *S. haematobium* and *S. manosni* control, which serve to deal with imported schistosomiasis cases brought into China as a result of travel and migration from other countries. High-quality scientific and technological innovation will positively aid in reaching the schistosomiasis elimination targets by 2030.

## Supplementary Information

**Additional file 1.** Valid patents for schistosomiasis control and prevention filed by Chinese applicants

**Additional file 2.** The number of patent applications between 1968 to 2020

## Data Availability

The datasets analysed during the current study are available from the corresponding author upon reasonable request.

## Abbreviations

WHO: World Health Organization
PCT: Patent Cooperation Treaty
GST: Glutathione-*S*-transferase
GR: Glutathione reductase
WNT: Wingless/integrated
ELISA: Enzyme-linked immunosorbent assay
LAMP: Loop-mediated isothermal amplification
PCR: Polymerase chain reaction
RPA: Recombinase polymerase amplification
GICA: Colloidal gold immunochromatographic assay
IHA: Indirect haemagglutination assays
DDIA: Dye dipstick immunoassay
DIGFA: Dot immunogold filtration assay
IPC: International Patent Classification
R&D: Research and Development
DNA: Deoxyribose nucleic acid
RNA: Ribonucleic acid
TDR: Special Programme for Research and Training in Tropical Diseases
