# Supplementary material for: Patented technologies for schistosomiasis control and prevention filed by Chinese applicants
Source: Infect Dis Poverty. 2021 Jun 12;10:84. doi: 10.1186/s40249-021-00869-6 (PMC8199835; doi:10.1186/s40249-021-00869-6)
Supplement: Supplementary file 1 — Additional file 1. Valid patents for schistosomiasis control and prevention filed by Chinese applicants [file 40249_2021_869_MOESM1_ESM.docx]

[Additional file1](https://static-content.springer.com/esm/art%3A10.1186%2Fs40249-017-0271-9/MediaObjects/40249_2017_271_MOESM1_ESM.pdf)

**Valid patents for schistosomiasis control and prevention filed by Chinese applicants**

**(By December 30, 2020)**

| **No.** | **Application date** | **Application number** | **Authorization code** | **Patent type** | **Patent title** | **Weblink** |
| --- | --- | --- | --- | --- | --- | --- |
|  | 20041016 | CN200410065018.6 | CN1251591C | Invention | Niclosamide ethanolamine salt powder and preparation method thereof | http://www.patexplorer.com/patent/view.html?patid=CN200410065018.6&q=id:CN200410065018.6 |
|  | 20041213 | CN200410066055.9 | CN100408126C | Invention | Production method and application of epidermal needle | http://www.patexplorer.com/patent/view.html?patid=CN200410066055.9&q=id:CN200410066055.9 |
|  | 20050616 | CN200510018929.8 | CN1290412C | Invention | The pharmaceutical composition for eliminating schistosomes and preparation method | http://www.patexplorer.com/patent/view.html?patid=CN200510018929.8&q=id:CN200510018929.8 |
|  | 20050825 | CN200510093030.2 | CN1302709C | Invention | Application of calcium cyanamide in preventing schistosomiasis | http://www.patexplorer.com/patent/view.html?patid=CN200510093030.2&q=id:CN200510093030.2 |
|  | 20051020 | CN200510030684.0 | CN100497627 | Invention | Cloning, expression, and application of antigen gene of *Schistosoma japonicum* vaccine | http://www.patexplorer.com/patent/view.html?patid=CN200510030684.0&q=id:CN200510030684.0 |
|  | 20060106 | CN200610005300.4 | CN100556890 | Invention | A kind of molluscacide | http://www.patexplorer.com/patent/view.html?patid=CN200610005300.4&q=id:CN200610005300.4 |
|  | 20061227 | CN200610136957.4 | CN101209043B | Invention | Application of sanguinarine or chelidonine in the control of schistosomiasis | http://www.patexplorer.com/patent/view.html?patid=CN200610136957.4&q=id:CN200610136957.4 |
|  | 20070125 | CN200710019447.3 | CN100455194C | Invention | A kind of molluscacide-niclosamide ethanolamine salt compound suspending agent and preparation method thereof | http://www.patexplorer.com/patent/view.html?patid=CN200710019447.3&q=id:CN200710019447.3 |
|  | 20070904 | CN200710053121.2 | CN101131347B | Invention | Fluorescence quantitative PCR kit for rapid detection of *Schistosoma japonicum* | http://www.patexplorer.com/patent/view.html?patid=CN200710053121.2&q=id:CN200710053121.2 |
|  | 20080315 | CN200810086500.6 | CN101238819B | Invention | Calcium cyanamide is used to kill schistosome eggs | http://www.patexplorer.com/patent/view.html?patid=CN200810086500.6&q=id:CN200810086500.6 |
|  | 20080422 | CN200810036422.9 | CN101565705B | Invention | *Schistosoma japonicum* microsatellite locus and its application | http://www.patexplorer.com/patent/view.html?patid=CN200810036422.9&q=id:CN200810036422.9 |
|  | 20080430 | CN200810036878.5 | CN101570573B | Invention | Method and kit for diagnosing schistosomiasis japonica | http://www.patexplorer.com/patent/view.html?patid=CN200810036878.5&q=id:CN200810036878.5 |
|  | 20080618 | CN200810031528.X | CN101327211B | Invention | Application of allocryptine and its salt in anti-liver fibrosis | http://www.patexplorer.com/patent/view.html?patid=CN200810031528.X&q=id:CN200810031528.X |
|  | 20080618 | CN200810031530.7 | CN101297870B | Invention | Application of total alkaloids or salt of Boluohui in anti-schistosoma-induced liver fibrosis | http://www.patexplorer.com/patent/view.html?patid=CN200810031530.7&q=id:CN200810031530.7 |
|  | 20080926 | CN200810043808.2 | CN101685097B | Invention | ELISA kit for detecting *Schistosoma japonicum* antibody and its detection method and application | http://www.patexplorer.com/patent/view.html?patid=CN200810043808.2&q=id:CN200810043808.2 |
|  | 20081111 | CN200810202535.1 | CN101736003B | Invention | *Schistosoma japonicum* miRNA and its application | http://www.patexplorer.com/patent/view.html?patid=CN200810202535.1&q=id:CN200810202535.1 |
|  | 20081128 | CN200810162767.9 | CN101433730B | Invention | Domestic garbage treatment agent containing plant vinegar liquid and its use | http://www.patexplorer.com/patent/view.html?patid=CN200810162767.9&q=id:CN200810162767.9 |
|  | 20090807 | CN200910060300.8 | CN101620226B | Invention | Kit for rapid determination of schistosomiasis and preparation method thereof | http://www.patexplorer.com/patent/view.html?patid=CN200910060300.8&q=id:CN200910060300.8 |
|  | 20090911 | CN200910066130.4 | CN101643750B | Invention | Construction method and application of targeted tumor-lytic adenovirus vector Ad-TD-gene | http://www.patexplorer.com/patent/view.html?patid=CN200910066130.4&q=id:CN200910066130.4 |
|  | 20091215 | CN200910250533.4 | CN101716319B | Invention | Traditional Chinese medicine composition for treating liver fibrosis, and preparation method and use thereof | http://www.patexplorer.com/patent/view.html?patid=CN200910250533.4&q=id:CN200910250533.4 |
|  | 20100325 | CN201010133142.7 | CN101921325B | Invention | An antigen for increasing CD4 + CD25 + Foxp3 + regulatory T cells and its application | http://www.patexplorer.com/patent/view.html?patid=CN201010133142.7&q=id:CN201010133142.7 |
|  | 20100402 | CN201010155316.X | CN102210314B | Invention | Molluscicidal composition containing spirolamide and carbaryl | http://www.patexplorer.com/patent/view.html?patid=CN201010155316.X&q=id:CN201010155316.X |
|  | 20100715 | CN201010224645.5 | CN101892239B | Invention | *Schistosoma japonicum* Frizzled9 gene, protein and use | http://www.patexplorer.com/patent/view.html?patid=CN201010224645.5&q=id:CN201010224645.5 |
|  | 20100716 | CN201010236532.7 | CN102010861B | Invention | Application of *Schistosoma japonicum* transposon DNA target sequence in diagnosis of schistosomiasis | http://www.patexplorer.com/patent/view.html?patid=CN201010236532.7&q=id:CN201010236532.7 |
|  | 20100930 | CN201010501767.4 | CN102000323B | Invention | Application of SJ16 protein in preparing immunosuppressive drugs | http://www.patexplorer.com/patent/view.html?patid=CN201010501767.4&q=id:CN201010501767.4 |
|  | 20100930 | CN201010501785.2 | CN101985468B | Invention | SJ16 recombinant protein and its application in preparing schistosomiasis vaccines, diagnostic reagents and therapeutic drugs | http://www.patexplorer.com/patent/view.html?patid=CN201010501785.2&q=id:CN201010501785.2 |
|  | 20101217 | CN201010593579.9 | CN102047880B | Invention | Niclosamide film spreading oil agent and preparation method and application thereof | http://www.patexplorer.com/patent/view.html?patid=CN201010593579.9&q=id:CN201010593579.9 |
|  | 20101231 | CN201010624797.4 | CN102078389B | Invention | Application of pulsatilla extract in preparation of anti-schistosomiasis medicine, preparation method of pulsatilla extract and preparation method of its preparation | http://www.patexplorer.com/patent/view.html?patid=CN201010624797.4&q=id:CN201010624797.4 |
|  | 20101231 | CN201010624574.8 | CN102068445B | Invention | Application of pulsatilla saponins in preparation of anti-schistosomiasis medicine and preparation method of the same | http://www.patexplorer.com/patent/view.html?patid=CN201010624574.8&q=id:CN201010624574.8 |
|  | 20110120 | CN201110022176.3 | CN102607888B | Invention | Automatic collection and real-time monitoring device for schistosome cercariae | http://www.patexplorer.com/patent/view.html?patid=CN201110022176.3&q=id:CN201110022176.3 |
|  | 20110120 | CN201110022202.2 | CN102600761B | Invention | A surface active substance based on N-(4-nitrophenyl)-5-chlorosalicylamide and its preparation technology | http://www.patexplorer.com/patent/view.html?patid=CN201110022202.2&q=id:CN201110022202.2 |
|  | 20110223 | CN201120045012.8 | CN201952786U | Utility model | Slotted floating type screw blocking device | http://www.patexplorer.com/patent/view.html?patid=CN201120045012.8&q=id:CN201120045012.8 |
|  | 20110311 | CN201120063159.X | CN201986589U | Utility model | Intermediate water intake and anti-*oncomelania* block | http://www.patexplorer.com/patent/view.html?patid=CN201120063159.X&q=id:CN201120063159.X |
|  | 20110418 | CN201110096964.7 | CN102217594B | Invention | A kind of molluscacide for *Oncomelania* snail-tetraacetaldehyde powder and preparation method thereof | http://www.patexplorer.com/patent/view.html?patid=CN201110096964.7&q=id:CN201110096964.7 |
|  | 20110418 | CN201110097790.6 | CN102217598B | Invention | Composition containing spironilamine and tetraacetaldehyde and its compound suspending agent | http://www.patexplorer.com/patent/view.html?patid=CN201110097790.6&q=id:CN201110097790.6 |
|  | 20110711 | CN201110193322.9 | CN102876682B | Invention | *Schistosoma japonicum* polymorphism antigen gene and its use as vaccine | http://www.patexplorer.com/patent/view.html?patid=CN201110193322.9&q=id:CN201110193322.9 |
|  | 20110722 | CN201110207371.3 | CN102273456B | Invention | Application of β-m-dihydroxybenzoic acid macrolide derivative in the prevention and control of harmful snails | http://www.patexplorer.com/patent/view.html?patid=CN201110207371.3&q=id:CN201110207371.3 |
|  | 20110722 | CN201110207375.1 | CN102265877B | Invention | Use of β-resoric acid macrolide in prevention and control of harmful snails | http://www.patexplorer.com/patent/view.html?patid=CN201110207375.1&q=id:CN201110207375.1 |
|  | 20110815 | CN201110232776.2 | CN102277373B | Invention | *Schistosoma japonicum* vaccine expression vector secreted and expressed by attenuated salmonella and application thereof | http://www.patexplorer.com/patent/view.html?patid=CN201110232776.2&q=id:CN201110232776.2 |
|  | 20111021 | CN201110322599.7 | CN103059117B | Invention | High-throughput screening of important antigens of *Schistosoma japonicum* and its application in the diagnosis of schistosomiasis | http://www.patexplorer.com/patent/view.html?patid=CN201110322599.7&q=id:CN201110322599.7 |
|  | 20111114 | CN201110359988.7 | CN103102404B | Invention | *Schistosoma japonicum* SjSap recombinant antigen protein and preparation method and application thereof | http://www.patexplorer.com/patent/view.html?patid=CN201110359988.7&q=id:CN201110359988.7 |
|  | 20120217 | CN201210036790.X | CN102631360B | Invention | Application of oleanane-type saponins in preparing medicines for treating and/or preventing schistosomiasis | http://www.patexplorer.com/patent/view.html?patid=CN201210036790.X&q=id:CN201210036790.X |
|  | 20120217 | CN201210036808.6 | CN102631361B | Invention | Application of oleanane-type saponins in preparing medicines for treating and/or preventing schistosomiasis | http://www.patexplorer.com/patent/view.html?patid=CN201210036808.6&q=id:CN201210036808.6 |
|  | 20120331 | CN201210094426.9 | CN103356518B | Invention | Use of monacillin β-resorate macrolide in the prevention and control of schistosomiasis | <http://www.patexplorer.com/patent/view.html?patid=CN201210094426.9&q=id:CN201210094426.9> |
|  | 20120821 | CN201210299103.3 | CN103622977B | Invention | Application of a compound in preparation of anti-schistosomiasis medicine | http://www.patexplorer.com/patent/view.html?patid=CN201210299103.3&q=id:CN201210299103.3 |
|  | 20121227 | CN201210580499.9 | CN103076447B | Invention | Schistosoma egg crude antigen purification method, related purified antigen and schistosome antibody detection colloidal gold immune kit | http://www.patexplorer.com/patent/view.html?patid=CN201210580499.9&q=id:CN201210580499.9 |
|  | 20121227 | CN201210579756.7 | CN103891720B | Invention | A Compound molluscacide composition containing snail-carbamate | http://www.patexplorer.com/patent/view.html?patid=CN201210579756.7&q=id:CN201210579756.7 |
|  | 20130109 | CN201310007886.8 | CN103910725B | Invention | A class of praziquantel analogs, preparation method and use thereof | http://www.patexplorer.com/patent/view.html?patid=CN201310007886.8&q=id:CN201310007886.8 |
|  | 20130407 | CN201310118394.6 | CN103197059B | Invention | Schistosomiasis electrochemical sensor rapid determination kit, detection method and preparation method thereof | http://www.patexplorer.com/patent/view.html?patid=CN201310118394.6&q=id:CN201310118394.6 |
|  | 20130509 | CN201320246796.X | CN203203856U | Utility model | *Oncomelania* crusher | http://www.patexplorer.com/patent/view.html?patid=CN201320246796.X&q=id:CN201320246796.X |
|  | 20130624 | CN201310255096.1 | CN104230743B | Invention | A kind of preparation method of 4-benzyl-1-phenethylpiperazine-2,6-dione | http://www.patexplorer.com/patent/view.html?patid=CN201310255096.1&q=id:CN201310255096.1 |
|  | 20130716 | CN201310298298.4 | CN104003954B | Invention | A class of 1,2,5-oxadiazole-2 oxide analogues, preparation method and use thereof | http://www.patexplorer.com/patent/view.html?patid=CN201310298298.4&q=id:CN201310298298.4 |
|  | 20130718 | CN201310304323.5 | CN103405779B | Invention | Long-acting artesunate medicine for preventing schistosomiasis infection and preparation method thereof | http://www.patexplorer.com/patent/view.html?patid=CN201310304323.5&q=id:CN201310304323.5 |
|  | 20130917 | CN201310423738.4 | CN103535377B | Invention | Preparation method of HAP/ZnO inorganic nano composite molluscicide | http://www.patexplorer.com/patent/view.html?patid=CN201310423738.4&q=id:CN201310423738.4 |
|  | 20131211 | CN201310676706.5 | CN104710323B | Invention | Salicylamide ester compound with molluscicidal activity and its preparation and use | http://www.patexplorer.com/patent/view.html?patid=CN201310676706.5&q=id:CN201310676706.5 |
|  | 20131226 | CN201310729167.7 | CN103735825B | Invention | Comprehensive conditioning traditional Chinese medicine composition and its application | http://www.patexplorer.com/patent/view.html?patid=CN201310729167.7&q=id:CN201310729167.7 |
|  | 20140109 | CN201410009408.5 | CN103724414B | Invention | A SjHSP90 Recombinant Protein and Its Application in Diagnosis and Curative Effect Evaluation of Schistosomiasis | http://www.patexplorer.com/patent/view.html?patid=CN201410009408.5&q=id:CN201410009408.5 |
|  | 20140714 | CN201410332494.3 | CN104074175B | Invention | Semi-open type swirling screw discharging device | http://www.patexplorer.com/patent/view.html?patid=CN201410332494.3&q=id:CN201410332494.3 |
|  | 20140729 | CN201410285116.4 | CN104047061B | Invention | Anti-*Schistosoma japonicum* thioredoxin glutathione reductase Sj TGR single domain antibody and preparation method thereof | http://www.patexplorer.com/patent/view.html?patid=CN201410285116.4&q=id:CN201410285116.4 |
|  | 20140730 | CN201410369500.2 | CN104133448B | Invention | Intelligent system for monitoring oncomelania and snail living environment based on wireless sensor network | http://www.patexplorer.com/patent/view.html?patid=CN201410369500.2&q=id:CN201410369500.2 |
|  | 20140825 | CN201410421620.2 | CN105343397B | Invention | Betel nut cataplasm, its preparation method and application | http://www.patexplorer.com/patent/view.html?patid=CN201410421620.2&q=id:CN201410421620.2 |
|  | 20141014 | CN201410542075.2 | CN105566475B | Invention | *Schistosoma japonicum* recombinant protein and its preparation method and application | http://www.patexplorer.com/patent/view.html?patid=CN201410542075.2&q=id:CN201410542075.2 |
|  | 20141110 | CN201410627554.4 | CN104318842B | Invention | High similarity simulated *oncomelania* and preparation method thereof | http://www.patexplorer.com/patent/view.html?patid=CN201410627554.4&q=id:CN201410627554.4 |
|  | 20141126 | CN201420732015.2 | CN204324946U | Utility model | Ultrasonic device for killing schistosome larvae | http://www.patexplorer.com/patent/view.html?patid=CN201420732015.2&q=id:CN201420732015.2 |
|  | 20141128 | CN201410708210.6 | CN105686892B | Invention | Mouse fixing device for schistosoma infection experiment and experiment method | http://www.patexplorer.com/patent/view.html?patid=CN201410708210.6&q=id:CN201410708210.6 |
|  | 20141204 | CN201410726505.6 | CN104531557B | Invention | A strain of Streptomyces subfrescens producing molluscicidal active substance and application thereof | http://www.patexplorer.com/patent/view.html?patid=CN201410726505.6&q=id:CN201410726505.6 |
|  | 20141204 | CN201410726401.5 | CN104498396B | Invention | A strain of Streptomyces griseus producing molluscicidal active substance and its application | http://www.patexplorer.com/patent/view.html?patid=CN201410726401.5&q=id:CN201410726401.5 |
|  | 20141204 | CN201410726301.2 | CN104388362B | Invention | A strain of black streptomyces griseus producing molluscicidal active substance and application thereof | http://www.patexplorer.com/patent/view.html?patid=CN201410726301.2&q=id:CN201410726301.2 |
|  | 20141215 | CN201410776588.X | CN104557862B | Invention | Heterocyclic compounds with Wnt signaling pathway inhibitory activity | http://www.patexplorer.com/patent/view.html?patid=CN201410776588.X&q=id:CN201410776588.X |
|  | 20141216 | CN201410781275.3 | CN104651489B | Invention | Q-PCR primers, identification methods and kits for identifying snails infected by *Schistosoma japonicum* | http://www.patexplorer.com/patent/view.html?patid=CN201410781275.3&q=id:CN201410781275.3 |
|  | 20141222 | CN201410795870.2 | CN104650224B | Invention | A Nanobody Targeting SEA and Its Coding Sequence and Application | http://www.patexplorer.com/patent/view.html?patid=CN201410795870.2&q=id:CN201410795870.2 |
|  | 20150119 | CN201510024897.6 | CN104558138B | Invention | The expression and purification method and application of SjP40 protein of *Schistosoma japonicum* | http://www.patexplorer.com/patent/view.html?patid=CN201510024897.6&q=id:CN201510024897.6 |
|  | 20150119 | CN201710705296.0 | CN107417784B | Invention | Use of SjP40 protein of *Schistosoma japonicum* | http://www.patexplorer.com/patent/view.html?patid=CN201710705296.0&q=id:CN201710705296.0 |
|  | 20150126 | CN201520051593.4 | CN204418134U | Utility model | Full overflow swirling screw discharging device | http://www.patexplorer.com/patent/view.html?patid=CN201520051593.4&q=id:CN201520051593.4 |
|  | 20150128 | CN201510043756.9 | CN104630172B | Invention | A mutant of schistosome glutathione-S-transferase and its application | http://www.patexplorer.com/patent/view.html?patid=CN201510043756.9&q=id:CN201510043756.9 |
|  | 20150312 | CN201510110138.1 | CN104663663B | Invention | Compound pesticide preparation containing spirocarb and niclosamide ethanolammonium salt and application | http://www.patexplorer.com/patent/view.html?patid=CN201510110138.1&q=id:CN201510110138.1 |
|  | 20150317 | CN201510117214.1 | CN104726585B | Invention | A detection method of artesunate acting on *Schistosoma japonicum* microRNA | http://www.patexplorer.com/patent/view.html?patid=CN201510117214.1&q=id:CN201510117214.1 |
|  | 20150408 | CN201510163554.8 | CN104876912B | Invention | Wnt signal pathway inhibitor and its application | http://www.patexplorer.com/patent/view.html?patid=CN201510163554.8&q=id:CN201510163554.8 |
|  | 20150502 | CN201510216181.6 | CN104782495B | Invention | A method of tissue culture and rapid propagation | http://www.patexplorer.com/patent/view.html?patid=CN201510216181.6&q=id:CN201510216181.6 |
|  | 20150616 | CN201510335088.7 | CN104962560B | Invention | A nucleic acid aptamer for detecting *Schistosoma japonicum* eggs and its application in preparing detection preparations | http://www.patexplorer.com/patent/view.html?patid=CN201510335088.7&q=id:CN201510335088.7 |
|  | 20150626 | CN201510362361.5 | CN104984334B | Invention | A kind of rabies attenuated vaccine-praziquantel compound and its preparation method and application | http://www.patexplorer.com/patent/view.html?patid=CN201510362361.5&q=id:CN201510362361.5 |
|  | 20150714 | CN201510413561.9 | CN104940229B | Invention | Composition capable of preventing sunscreen and preventing malaria, trypanosomiasis and schistosomiasis | http://www.patexplorer.com/patent/view.html?patid=CN201510413561.9&q=id:CN201510413561.9 |
|  | 20150728 | CN201520554886.4 | CN204969008U | Utility model | Schistosomiasis detection device | http://www.patexplorer.com/patent/view.html?patid=CN201520554886.4&q=id:CN201520554886.4 |
|  | 20150808 | CN201510480266.5 | CN105061515B | Invention | Synthesis of a phosphorescent iridium complex and its use in fluorescent labeling of cercariae of schistosomiasis | http://www.patexplorer.com/patent/view.html?patid=CN201510480266.5&q=id:CN201510480266.5 |
|  | 20150821 | CN201510519076.X | CN105153290B | Invention | *Schistosoma japonicum* SjCTRL recombinant antigen protein and preparation method and application thereof | http://www.patexplorer.com/patent/view.html?patid=CN201510519076.X&q=id:CN201510519076.X |
|  | 20150906 | CN201510560256.2 | CN105256759B | Invention | A comprehensive method for preventing and eliminating snails and snails in horizontal stratified ecological interference of beaches | http://www.patexplorer.com/patent/view.html?patid=CN201510560256.2&q=id:CN201510560256.2 |
|  | 20150906 | CN201520684149.6 | CN204930089U | Utility model | Liquid snail killing drug delivery device | http://www.patexplorer.com/patent/view.html?patid=CN201520684149.6&q=id:CN201520684149.6 |
|  | 20150917 | CN201510595048.6 | CN105123698B | Invention | Compound pesticide preparation containing spirocarb and niacinanilide and application | http://www.patexplorer.com/patent/view.html?patid=CN201510595048.6&q=id:CN201510595048.6 |
|  | 20150929 | CN201510630548.9 | CN105078963B | Invention | Application of α-tocopherol in preparation of medicine for treating schistosomiasis | http://www.patexplorer.com/patent/view.html?patid=CN201510630548.9&q=id:CN201510630548.9 |
|  | 20151016 | CN201520803196.8 | CN205040488U | Utility model | Electric control screw device for controlling the spread of *Oncomelania* snails and *Schistosoma* in ditch by using electric current | http://www.patexplorer.com/patent/view.html?patid=CN201520803196.8&q=id:CN201520803196.8 |
|  | 20151027 | CN201510706078.X | CN106608917B | Invention | *Schistosoma japonicum* recombinant multi-epitope antigen and its application | http://www.patexplorer.com/patent/view.html?patid=CN201510706078.X&q=id:CN201510706078.X |
|  | 20151103 | CN201510733132.X | CN105230523B | Invention | Sheep dung collection device and use method thereof | http://www.patexplorer.com/patent/view.html?patid=CN201510733132.X&q=id:CN201510733132.X |
|  | 20151117 | CN201510790100.3 | CN105394037B | Invention | Niclosamide ethanolamine salt self-emulsifying microemulsion and its preparation method | http://www.patexplorer.com/patent/view.html?patid=CN201510790100.3&q=id:CN201510790100.3 |
|  | 20151123 | CN201510818639.5 | CN105384803B | Invention | A *Schistosoma japonicum* recombinant protein SjSAPLP4 and its coding gene and application | http://www.patexplorer.com/patent/view.html?patid=CN201510818639.5&q=id:CN201510818639.5 |
|  | 20151123 | CN201510818349.0 | CN105254732B | Invention | A *Schistosoma japonicum* recombinant protein SjSAPLP5 and its coding gene and application | http://www.patexplorer.com/patent/view.html?patid=CN201510818349.0&q=id:CN201510818349.0 |
|  | 20151127 | CN201510853180.2 | CN105663134B | Invention | Medicinal composition for preventing and treating acute schistosomiasis infection | http://www.patexplorer.com/patent/view.html?patid=CN201510853180.2&q=id:CN201510853180.2 |
|  | 20151130 | CN201520968186.X | CN205233203U | Utility model | Schistosoma miracidium observation device | http://www.patexplorer.com/patent/view.html?patid=CN201520968186.X&q=id:CN201520968186.X |
|  | 20151207 | CN201510889423.8 | CN105330602B | Invention | A kind of decoquinate analogue and its application | http://www.patexplorer.com/patent/view.html?patid=CN201510889423.8&q=id:CN201510889423.8 |
|  | 20151215 | CN201510931300.6 | CN105385686B | Invention | *Schistosoma japonicum* nucleic acid detection kit and detection method based on improved LAMP method | http://www.patexplorer.com/patent/view.html?patid=CN201510931300.6&q=id:CN201510931300.6 |
|  | 20151223 | CN201510979220.8 | CN105435230B | Invention | Application of O-GlcNAc glycosylation modification in the prevention and control of liver fibrosis | http://www.patexplorer.com/patent/view.html?patid=CN201510979220.8&q=id:CN201510979220.8 |
|  | 20160105 | CN201620007042.2 | CN205352728U | Utility model | *Schistosoma japonicum* cercariae bionic collector | http://www.patexplorer.com/patent/view.html?patid=CN201620007042.2&q=id:CN201620007042.2 |
|  | 20160127 | CN201610056643.7 | CN105648059B | Invention | RPA-based *Schistosoma japonicum* nucleic acid detection kit and detection method | http://www.patexplorer.com/patent/view.html?patid=CN201610056643.7&q=id:CN201610056643.7 |
|  | 20160129 | CN201610060560.5 | CN105601675B | Invention | Synthesis of a class of phosphorescent iridium complexes and their fluorescent labeling of adult schistosome | http://www.patexplorer.com/patent/view.html?patid=CN201610060560.5&q=id:CN201610060560.5 |
|  | 20160309 | CN201610132093.2 | CN105638690B | Invention | Pesticide composition containing emamectin benzoate and spironamide ethanolammonium salt | http://www.patexplorer.com/patent/view.html?patid=CN201610132093.2&q=id:CN201610132093.2 |
|  | 20160314 | CN201610144315.2 | CN105699379B | Invention | Schistosoma mircaria detection device | http://www.patexplorer.com/patent/view.html?patid=CN201610144315.2&q=id:CN201610144315.2 |
|  | 20160314 | CN201610144116.1 | CN105787460B | Invention | Automatic identification method of Schistosoma miracidium | http://www.patexplorer.com/patent/view.html?patid=CN201610144116.1&q=id:CN201610144116.1 |
|  | 20160329 | CN201620251070.9 | CN205449619U | Utility model | *Oncomelania* crusher | http://www.patexplorer.com/patent/view.html?patid=CN201620251070.9&q=id:CN201620251070.9 |
|  | 20160503 | CN201610282900.9 | CN105816421B | Invention | Praziquantel nanoemulsion in-situ gel for preventing and treating schistosomiasis, and preparation method and application thereof | http://www.patexplorer.com/patent/view.html?patid=CN201610282900.9&q=id:CN201610282900.9 |
|  | 20160602 | CN201610387210.X | CN106093036B | Invention | Rotary Schistosoma cercariae enrichment and imaging device | http://www.patexplorer.com/patent/view.html?patid=CN201610387210.X&q=id:CN201610387210.X |
|  | 20160607 | CN201620554391.6 | CN205830785U | Utility model | A snail inspection frame for *oncomelania s*urvey within the ground diameter of the plant in the nursery stock planting park | http://www.patexplorer.com/patent/view.html?patid=CN201620554391.6&q=id:CN201620554391.6 |
|  | 20160721 | CN201620772511.X | CN205833239U | Utility model | *Oncomelania* smashing device | http://www.patexplorer.com/patent/view.html?patid=CN201620772511.X&q=id:CN201620772511.X |
|  | 20160721 | CN201620777052.4 | CN206008797U | Utility model | Automatic snail crushing device | http://www.patexplorer.com/patent/view.html?patid=CN201620777052.4&q=id:CN201620777052.4 |
|  | 20160817 | CN201610675071.0 | CN106280533B | Invention | A near-infrared fluorescent dye and its synthesis method and its use in fluorescent marking of parasites | http://www.patexplorer.com/patent/view.html?patid=CN201610675071.0&q=id:CN201610675071.0 |
|  | 20160831 | CN201610766923.7 | CN106172262B | Invention | Semi-automatic animal dung *Schistosoma japonicum* egg hatching device and hatching method thereof | http://www.patexplorer.com/patent/view.html?patid=CN201610766923.7&q=id:CN201610766923.7 |
|  | 20160926 | CN201610850360.X | CN106565673B | Invention | 5-fluoropyrimidine heterocyclic compound with Wnt signal pathway inhibitory activity and application thereof | http://www.patexplorer.com/patent/view.html?patid=CN201610850360.X&q=id:CN201610850360.X |
|  | 20160926 | CN201610850357.8 | CN107286135B | Invention | Heterocyclic compound with Wnt signal pathway inhibitory activity and application thereof | http://www.patexplorer.com/patent/view.html?patid=CN201610850357.8&q=id:CN201610850357.8 |
|  | 20160926 | CN201610850358.2 | CN107286136B | Invention | A 3-fluoropyridine heterocyclic compound and its application | http://www.patexplorer.com/patent/view.html?patid=CN201610850358.2&q=id:CN201610850358.2 |
|  | 20161011 | CN201610886194.9 | CN106244585B | Invention | A simple and efficient method for extracting mitochondrial genomic DNA from *Oncomelania* hupensis | http://www.patexplorer.com/patent/view.html?patid=CN201610886194.9&q=id:CN201610886194.9 |
|  | 20161027 | CN201610969748.1 | CN106947804B | Invention | Method for detecting schistosomiasis infective snails and kit and primers used in the method | http://www.patexplorer.com/patent/view.html?patid=CN201610969748.1&q=id:CN201610969748.1 |
|  | 20161102 | CN201621171143.X | CN206223634U | Utility model | Schistosoma cercariae in vivo imaging detection equipment | http://www.patexplorer.com/patent/view.html?patid=CN201621171143.X&q=id:CN201621171143.X |
|  | 20161102 | CN201621170425.8 | CN206177815U | Utility model | Fluorescence detection device for schistosome cercariae | http://www.patexplorer.com/patent/view.html?patid=CN201621170425.8&q=id:CN201621170425.8 |
|  | 20161116 | CN201611006487.X | CN106632142B | Invention | A 1,2,5- Thiadiazole- 2 oxide analogue and its application | http://www.patexplorer.com/patent/view.html?patid=CN201611006487.X&q=id:CN201611006487.X |
|  | 20161130 | CN201621295639.8 | CN206576979U | Utility model | Oral drug delivery device for dogs | http://www.patexplorer.com/patent/view.html?patid=CN201621295639.8&q=id:CN201621295639.8 |
|  | 20161130 | CN201621295715.5 | CN206659961U | Utility model | Cattle oral drug delivery device | http://www.patexplorer.com/patent/view.html?patid=CN201621295715.5&q=id:CN201621295715.5 |
|  | 20161130 | CN201621295638.3 | CN206659960U | Utility model | Sheep oral drug delivery device | http://www.patexplorer.com/patent/view.html?patid=CN201621295638.3&q=id:CN201621295638.3 |
|  | 20161209 | CN201621348111.2 | CN206350443U | Utility model | Device for automatically dripping and killing *oncomelania* drugs | http://www.patexplorer.com/patent/view.html?patid=CN201621348111.2&q=id:CN201621348111.2 |
|  | 20170122 | CN201710047340.3 | CN106645352B | Invention | Nano antibody composition for detecting schistosomiasis, immunosensor and preparation method and application thereof | http://www.patexplorer.com/patent/view.html?patid=CN201710047340.3&q=id:CN201710047340.3 |
|  | 20170207 | CN201710067858.3 | CN106701985B | Invention | A multiple DPO-PCR primer combination and method for detecting Trichinella spiralis, Toxoplasma gondii and Schistosoma | http://www.patexplorer.com/patent/view.html?patid=CN201710067858.3&q=id:CN201710067858.3 |
|  | 20170222 | CN201710095258.8 | CN106895987B | Invention | Animal skin carrier for obtaining *Schistosoma japonicum* cercariae and preparation method thereof | http://www.patexplorer.com/patent/view.html?patid=CN201710095258.8&q=id:CN201710095258.8 |
|  | 20170228 | CN201710115410.4 | CN106928274B | Invention | Diploid derivative of dihydroartemisinin, its pharmaceutical composition and application | http://www.patexplorer.com/patent/view.html?patid=CN201710115410.4&q=id:CN201710115410.4 |
|  | 20170516 | CN201710355972.6 | CN107183041B | Invention | Application of aldicarb in preparation of molluscicide | http://www.patexplorer.com/patent/view.html?patid=CN201710355972.6&q=id:CN201710355972.6 |
|  | 20170516 | CN201710356059.8 | CN107156123B | Invention | Application of Chlorothalonil in Preparation of *Oncomelania*-killing Medicine | http://www.patexplorer.com/patent/view.html?patid=CN201710356059.8&q=id:CN201710356059.8 |
|  | 20170516 | CN201710355973.0 | CN107183047B | Invention | Application of picoline in the preparation of *oncomelania* drugs | http://www.patexplorer.com/patent/view.html?patid=CN201710355973.0&q=id:CN201710355973.0 |
|  | 20170516 | CN201710355975.X | CN107087608B | Invention | Application of chlorophenoxyacetic acid in the preparation of *oncomelania* drugs | http://www.patexplorer.com/patent/view.html?patid=CN201710355975.X&q=id:CN201710355975.X |
|  | 20170522 | CN201720569139.7 | CN207118495U | Utility model | A schistosome egg separator | http://www.patexplorer.com/patent/view.html?patid=CN201720569139.7&q=id:CN201720569139.7 |
|  | 20170613 | CN201720686029.9 | CN206857244U | Utility model | Portable *Oncomelania* Box | http://www.patexplorer.com/patent/view.html?patid=CN201720686029.9&q=id:CN201720686029.9 |
|  | 20170613 | CN201720686445.9 | CN206851749U | Utility model | Folding snail survey frame | http://www.patexplorer.com/patent/view.html?patid=CN201720686445.9&q=id:CN201720686445.9 |
|  | 20170619 | CN201710463669.8 | CN107058595B | Invention | Schistosoma egg detection method and kit and primer used in the method | http://www.patexplorer.com/patent/view.html?patid=CN201710463669.8&q=id:CN201710463669.8 |
|  | 20170829 | CN201721091386.7 | CN207444043U | Utility model | Operation cabinet for incubation of fecal miracidia | http://www.patexplorer.com/patent/view.html?patid=CN201721091386.7&q=id:CN201721091386.7 |
|  | 20171019 | CN201721355444.2 | CN207699430U | Utility model | A biogas digester capable of killing schistosome eggs | http://www.patexplorer.com/patent/view.html?patid=CN201721355444.2&q=id:CN201721355444.2 |
|  | 20180111 | CN201820042449.8 | CN207689383U | Utility model | Schistosoma cercariae distribution detection equipment | http://www.patexplorer.com/patent/view.html?patid=CN201820042449.8&q=id:CN201820042449.8 |
|  | 20180111 | CN201820042368.8 | CN207689373U | Utility model | Schistosoma cercariae detection equipment for in vivo detection | http://www.patexplorer.com/patent/view.html?patid=CN201820042368.8&q=id:CN201820042368.8 |
|  | 20180111 | CN201820042447.9 | CN207689351U | Utility model | Equipment for detecting schistosome cercariae in infectious water bodies | http://www.patexplorer.com/patent/view.html?patid=CN201820042447.9&q=id:CN201820042447.9 |
|  | 20180209 | CN201810136573.5 | CN108344722B | Invention | Application of a near-infrared xanthene fluorescent dye in the fluorescent labeling of schistosome adults | http://www.patexplorer.com/patent/view.html?patid=CN201810136573.5&q=id:CN201810136573.5 |
|  | 20180211 | CN201810139449.4 | CN108299255B | Invention | A selective histone deacetylase 8 inhibitor and its preparation method and application | http://www.patexplorer.com/patent/view.html?patid=CN201810139449.4&q=id:CN201810139449.4 |
|  | 20180320 | CN201810230533.7 | CN108421601B | Invention | A new type of schistosomiasis control drug preparation device | http://www.patexplorer.com/patent/view.html?patid=CN201810230533.7&q=id:CN201810230533.7 |
|  | 20180320 | CN201810230153.3 | CN108480336B | Invention | Schistosomiasis kit device | http://www.patexplorer.com/patent/view.html?patid=CN201810230153.3&q=id:CN201810230153.3 |
|  | 20180320 | CN201810231668.5 | CN108405088B | Invention | Device for preparing medicine for preventing and treating schistosomiasis | http://www.patexplorer.com/patent/view.html?patid=CN201810231668.5&q=id:CN201810231668.5 |
|  | 20180321 | CN201820381357.2 | CN208532563U | Utility model | Non-polluted feces treatment equipment | http://www.patexplorer.com/patent/view.html?patid=CN201820381357.2&q=id:CN201820381357.2 |
|  | 20180415 | CN201820528885.6 | CN208144241U | Utility model | *Oncomelania* separation collection device | http://www.patexplorer.com/patent/view.html?patid=CN201820528885.6&q=id:CN201820528885.6 |
|  | 20180415 | CN201820528884.1 | CN208144240U | Utility model | Portable semi-automatic snail search machine | http://www.patexplorer.com/patent/view.html?patid=CN201820528884.1&q=id:CN201820528884.1 |
|  | 20180415 | CN201820528893.0 | CN208144242U | Utility model | An easy-to-operate snail collecting device | <http://www.patexplorer.com/patent/view.html?patid=CN201921268081.8&q=id:CN201921268081.8> |
|  | 20180516 | CN201820730827.1 | CN208274337U | Utility model | *Oncomelania* investigation tool backpack for prevention and control of schistosomiasis | http://www.patexplorer.com/patent/view.html?patid=CN201820730827.1&q=id:CN201820730827.1 |
|  | 20180516 | CN201820726819.X | CN208746540U | Utility model | Schistosomiasis pathogen detection kit | http://www.patexplorer.com/patent/view.html?patid=CN201820726819.X&q=id:CN201820726819.X |
|  | 20180516 | CN201820725986.2 | CN208175771U | Utility model | *Oncomelania* feeding device | http://www.patexplorer.com/patent/view.html?patid=CN201820725986.2&q=id:CN201820725986.2 |
|  | 20180612 | CN201820900473.0 | CN209352764U | Utility model | Fishing boat manure treatment system | http://www.patexplorer.com/patent/view.html?patid=CN201820900473.0&q=id:CN201820900473.0 |
|  | 20180630 | CN201821025787.7 | CN209437414U | Utility model | Neck sleeve deinsectization device for removing schistosomes on necks of dogs | http://www.patexplorer.com/patent/view.html?patid=CN201821025787.7&q=id:CN201821025787.7 |
|  | 20180703 | CN201821042648.5 | CN208633059U | Utility model | Ecological bank protection system for suppressing snails and blood | http://www.patexplorer.com/patent/view.html?patid=CN201821042648.5&q=id:CN201821042648.5 |
|  | 20180703 | CN201821043509.4 | CN208370484U | Utility model | A system for preventing and eliminating snails and beaches from ecological interference | http://www.patexplorer.com/patent/view.html?patid=CN201821043509.4&q=id:CN201821043509.4 |
|  | 20180713 | CN201810771342.1 | CN108517042B | Invention | Supramolecular hydrogel and preparation method thereof, and sustained-release preparation of schistosome cercariae and preparation method thereof | http://www.patexplorer.com/patent/view.html?patid=CN201810771342.1&q=id:CN201810771342.1 |
|  | 20180725 | CN201821186710.8 | CN208792454U | Utility model | Environmental protection toilet for ships | http://www.patexplorer.com/patent/view.html?patid=CN201821186710.8&q=id:CN201821186710.8 |
|  | 20180827 | CN201810981211.6 | CN109197896B | Invention | Plant-derived organic-inorganic compound molluscicidal fertilizer preparation and preparation method and application thereof | http://www.patexplorer.com/patent/view.html?patid=CN201810981211.6&q=id:CN201810981211.6 |
|  | 20180930 | CN201821621422.0 | CN212153263U | Utility model | A kind of snail removal project of navigable water diversion project | http://www.patexplorer.com/patent/view.html?patid=CN201821621422.0&q=id:CN201821621422.0 |
|  | 20181026 | CN201821749246.9 | CN209711569U | Utility model | Multifunctional vest applicable to on-site prevention and control of schistosomiasis | http://www.patexplorer.com/patent/view.html?patid=CN201821749246.9&q=id:CN201821749246.9 |
|  | 20181107 | CN201821827583.5 | CN209085662U | Utility model | Device for tracking *oncomelania* drifting track | http://www.patexplorer.com/patent/view.html?patid=CN201821827583.5&q=id:CN201821827583.5 |
|  | 20181229 | CN201811639058.5 | CN109619044B | Invention | Method for quickly preparing unisexual worms of *Schistosoma japonicum* | http://www.patexplorer.com/patent/view.html?patid=CN201811639058.5&q=id:CN201811639058.5 |
|  | 20190201 | CN201910103672.8 | CN109886170B | Invention | *Oncomelania* intelligent detection, identification and statistics system | http://www.patexplorer.com/patent/view.html?patid=CN201910103672.8&q=id:CN201910103672.8 |
|  | 20190624 | CN201910549543.1 | CN110330556B | Invention | Genes highly expressed in *Schistosoma japonicum* and their coding proteins and applications | http://www.patexplorer.com/patent/view.html?patid=CN201910549543.1&q=id:CN201910549543.1 |
|  | 20190624 | CN201910550310.3 | CN110305871B | Invention | Genes highly expressed in *Schistosoma japonicum* and their coding proteins and applications | http://www.patexplorer.com/patent/view.html?patid=CN201910550310.3&q=id:CN201910550310.3 |
|  | 20190627 | CN201910569736.3 | CN110227160B | Invention | Application of IRF4 gene in anti-schistosomiasis infection | http://www.patexplorer.com/patent/view.html?patid=CN201910569736.3&q=id:CN201910569736.3 |
|  | 20190711 | CN201910623065.4 | CN110243648B | Invention | Method for preparing schistosome egg specimens by using modified Kato thick smear | http://www.patexplorer.com/patent/view.html?patid=CN201910623065.4&q=id:CN201910623065.4 |
|  | 20190807 | CN201921268081.8 | CN210328957U | Utility model | A block for a batch of cercaria shedding | <http://www.patexplorer.com/patent/view.html?patid=CN201921268081.8&q=id:CN201921268081.8> |
|  | 20190815 | CN201921324220.4 | CN210737454U | Utility model | Ecological bank protection against *oncomelania* | http://www.patexplorer.com/patent/view.html?patid=CN201921324220.4&q=id:CN201921324220.4 |
|  | 20191016 | CN201921734173.0 | CN212017414U | Utility model | Device of a detection filter membrane for filtering *Schistosoma* *haematobium* eggs | http://www.patexplorer.com/patent/view.html?patid=CN201921734173.0&q=id:CN201921734173.0 |
|  | 20191101 | CN201921868365.0 | CN211322768U | Utility model | *Oncomelania* snail-preventing blocking net for middle-layer water intake | http://www.patexplorer.com/patent/view.html?patid=CN201921868365.0&q=id:CN201921868365.0 |
|  | 20191101 | CN201921868396.6 | CN211312446U | Utility model | *Oncomelania* wall and snail settlement tank for water conservancy and hydropower projects | <http://www.patexplorer.com/patent/view.html?patid=CN201921868396.6&q=id:CN201921868396.6> |
|  | 20191104 | CN201921875351.1 | CN211407325U | Utility model | Schistosomiasis detection and observation device for miracidia | http://www.patexplorer.com/patent/view.html?patid=CN201921875351.1&q=id:CN201921875351.1 |
|  | 20191129 | CN201922099792.3 | CN211153419U | Utility model | Grass curtain type snail trapping device | http://www.patexplorer.com/patent/view.html?patid=CN201922099792.3&q=id:CN201922099792.3 |
|  | 20191129 | CN201922113856.0 | CN210835419U | Utility model | Combined device for *oncomelania* biological imaging based on microscope and smartphone | http://www.patexplorer.com/patent/view.html?patid=CN201922113856.0&q=id:CN201922113856.0 |
|  | 20191206 | CN201922162620.6 | CN211407295U | Utility model | *Oncomelania* isolated feeding device | http://www.patexplorer.com/patent/view.html?patid=CN201922162620.6&q=id:CN201922162620.6 |
|  | 20191225 | CN201922370600.8 | CN211558609U | Utility model | Underwater nocturnal schistosome cercariae monitoring device | http://www.patexplorer.com/patent/view.html?patid=CN201922370600.8&q=id:CN201922370600.8 |
|  | 20200119 | CN202020115001.1 | CN211583644U | Utility model | Anatomy device suitable for mouse perfusion | http://www.patexplorer.com/patent/view.html?patid=CN202020115001.1&q=id:CN202020115001.1 |
|  | 20200303 | CN202020244413.5 | CN211652567U | Utility model | Automatic collection and imaging monitoring device for schistosome cercariae | http://www.patexplorer.com/patent/view.html?patid=CN202020244413.5&q=id:CN202020244413.5 |
|  | 20200520 | CN202020855397.3 | CN212164720U | Utility model | Individual separation device of schistosome mircaria specimens | http://www.patexplorer.com/patent/view.html?patid=CN202020855397.3&q=id:CN202020855397.3 |
