# Supplementary material for: Patented technologies for schistosomiasis control and prevention filed by Chinese applicants
Source: Infect Dis Poverty. 2021 Jun 12;10:84. doi: 10.1186/s40249-021-00869-6 (PMC8199835; doi:10.1186/s40249-021-00869-6)
Supplement: Supplementary file 2 — Additional file 2. The number of patent applications between 1968 to 2020 [file 40249_2021_869_MOESM2_ESM.docx]

[Additional file 2](https://static-content.springer.com/esm/art%3A10.1186%2Fs40249-017-0271-9/MediaObjects/40249_2017_271_MOESM1_ESM.pdf)

**The number of patent applications between 1968 to 2020**

| **Year** | **Number of patents** |
| --- | --- |
| 1968 | 1 |
| 1985 | 1 |
| 1986 | 0 |
| 1987 | 1 |
| 1988 | 0 |
| 1989 | 0 |
| 1990 | 0 |
| 1991 | 1 |
| 1992 | 8 |
| 1993 | 8 |
| 1994 | 7 |
| 1995 | 5 |
| 1996 | 2 |
| 1997 | 1 |
| 1998 | 8 |
| 1999 | 5 |
| 2000 | 8 |
| 2001 | 2 |
| 2002 | 9 |
| 2003 | 11 |
| 2004 | 22 |
| 2005 | 50 |
| 2006 | 29 |
| 2007 | 28 |
| 2008 | 46 |
| 2009 | 35 |
| 2010 | 37 |
| 2011 | 59 |
| 2012 | 47 |
| 2013 | 43 |
| 2014 | 46 |
| 2015 | 66 |
| 2016 | 59 |
| 2017 | 50 |
| 2018 | 71 |
| 2019 | 67 |
| 2020 | 26 |
